# Carbon stocks of above- and belowground tree biomass in Kibate Forest around Wonchi Crater Lake, Central Highland of Ethiopia

**DOI:** 10.1371/journal.pone.0254231

**Published:** 2021-07-09

**Authors:** Misganaw Meragiaw, Zerihun Woldu, Vegard Martinsen, Bal Ram Singh

**Affiliations:** 1 Department of Plant Biology & Biodiversity Management, College of Natural and Computational Sciences, Addis Ababa University, Addis Ababa, Ethiopia; 2 Faculty of Environmental Sciences and Natural Resource Management, Norwegian University of Life Sciences, Ås, Norway; Technical University in Zvolen, SLOVAKIA

## Abstract

Forests play an important role in the global carbon (C) balance, but their biomass has decreased globally mainly because of deforestation and a reduction in forest cover. However, little is known about the C stock of tree biomass related to environmental factors in the remnant forest patches. Thus, the present study aimed at assessing the status of C stocks of tree biomass using an allometric equation in Kibate Forest (Ethiopia). Sixty–six plots (30×30 m) were laid out at 100 m interval distance along the altitudinal gradients in five transects. The results revealed that the highest C stocks (67.4%) per species were contributed by *Juniperus procera*, *Ilex mitis* var. *mitis*, *Nuxia congesta*, and *Olea europaea* subsp. *cuspidata*. The mean total tree biomass was 91.9 ± 10.01 Mg ha^−1^. The mean total C stock was 45.9 ± 5.17 Mg ha^−1^, out of which 38.3 ± 4.31 and 7.7 ± 0.91 Mg ha^−1^ were stored in above- and belowground C pools, respectively. Anthropogenic factors were negatively associated with the C-stock distribution in the study area. Thus, the status of the C stock of tree biomass related to anthropogenic factors indicates that sustainable forest management practice is needed in the study area to conserve biodiversity and mitigate climate change.

## Introduction

Forests play an important role in the global carbon (C) balance for storage of C and hence combat adverse global climate change among other ecosystem services [1–7]. However, C stocks in forest biomass decreased globally mainly because of a reduction in the global forest cover. The C stock is decreasing at the rate of 1–2 billion Mg per year due to human activities in tropical and subtropical forests [5]. Deforestation and forest degradation typically account for 17–20% of the world’s greenhouse gas (GHG) emissions [1, 8–11]. In Africa, deforestation accounts for 70% of GHG emissions [12]. If deforestation continues at the present rate of 2%, about 2.76 billion Mg of C stored in forest vegetation in Ethiopia will be released to the atmosphere within 50 years [13–15]. Carbon dioxide (CO_2_), which is partly released as a result of forest degradation, contributes to about 60% of the anthropogenic greenhouse effects and climate change [16, 17]. Climate change, which is a major global challenge of our time, has many mounting pieces of evidence of irreversible environmental impacts [10, 16, 18]. The dilemma is that the rapid increment of CO_2_ concentration in the atmosphere is coupled with increasing human population and land-use changes [1, 6, 19–21]. Deforestation and land-use changes are serious problems in Sub-Saharan African countries [22, 23], both of which are contemporaneously leading natural forests into a state of progressive shrinkage in size [21, 24–26].

Forest C sequestration is an expanding research topic that addresses local and global strategies for the reduction of emissions of CO_2_ into the atmosphere [27, 28]. However, only a few studies have been quantifying C stocks in forests worldwide [6, 12, 29–36], and many forests are unexplored [37, 38]. Clear cutting is the most destructive and accurate method for the measurement of tree biomass, but it is environmentally unfriendly and time-consuming [6, 39, 40]. Thus, the nondestructive method using allometric equations is widely applicable in degraded forests for total carbon (TC) stock estimation [6, 12, 29–32, 37]. Allometric equations are statistical relationships between parameter standards for biomass of trees, such as diameter at breast height (DBH, cm), height (H, m), and wood density (ρ, g cm^−3^) [6, 41]. However, the tree height and wood density data on tropical species are lacking except for a few commercial timber species [42]. Thus, the tropical forest biomass has been estimated by applying the corresponding regression equations [8, 29, 32, 43, 44]. However, studies on C stocks of aboveground biomass (AGB) and belowground biomass (BGB) of trees related to environmental factors have been very limited in Ethiopia [45–48] and completely lacking in the study area. Assessments of the status of forest C stocks through estimation of AGB and BGB of trees are therefore urgently needed.

The assessment of the status of C stocks of tree biomass was conducted in Kibate Forest to address the following research questions: 1. Are there variations in accounting for the TC stock among tree species in Kibate Forest? 2. What does the distribution of TC stocks related to species richness look like? 3. Is there a significant relationship between the physical (altitude and slope) and anthropogenic (human intrusion and over-grazing by livestock) factors and C-stock distribution in Kibate Forest? Based on these research questions, the present study in Kibate Forest of Wonchi highland aimed at:

1. identifying the dominant tree species for TC stocks in Kibate Forest,
2. investigating the relationships between species richness and TC stocks,
3. examining the relationship between TC stocks and environmental variables such as altitude, slope, human impact, and overgrazing by livestock, and
4. assessing the status of C stocks of AGB and BGB trees in Kibate Forest.

## Materials and methods

### Description of the study area

**Fig 1:**
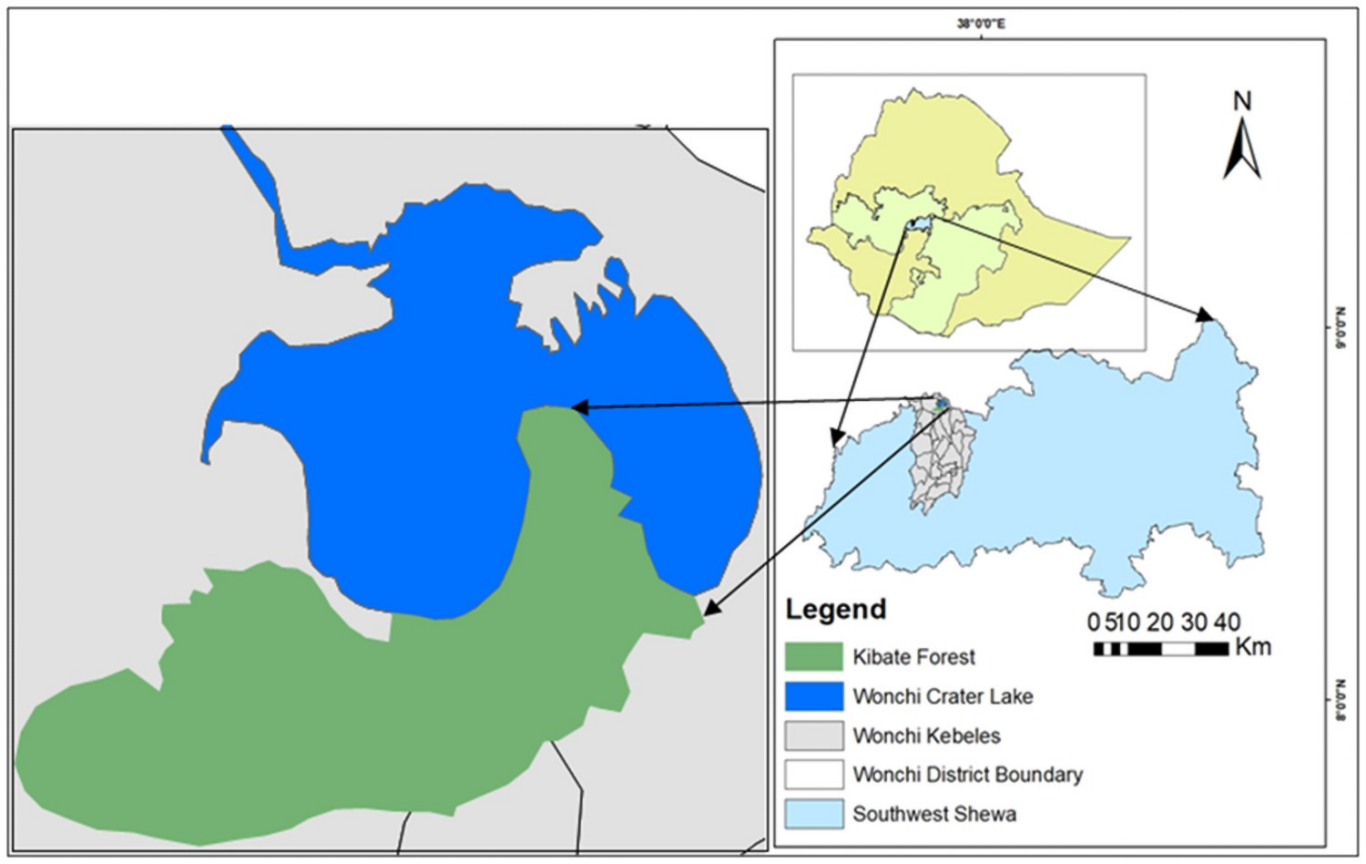
Map of Ethiopia showing the location of the study site. (Source: Adapted from Meragiaw et al. [49]).

The present study was carried out in Kibate Forest around Wonchi Crater Lake, Southwest Shewa Zone of the Central Highland of Ethiopia (Fig 1). Wonchi Crater Lake has recently been selected as a tourism project to construct a beautiful recreational center in the country through the ‘Dine for Nation Project’ program. Kibate Forest covers 450 ha with altitudinal ranges between 2800 and 3387 m a.s.l. [49]. The vegetation type of the study area is a typical dry evergreen montane forest [50]. Rapid forest degradation due to the expansion of farmland to steep and marginal areas in the district [26] could have contributed to high C emissions and soil degradation since effective C management practices have not been applied in the area [14].

**Fig 2:**
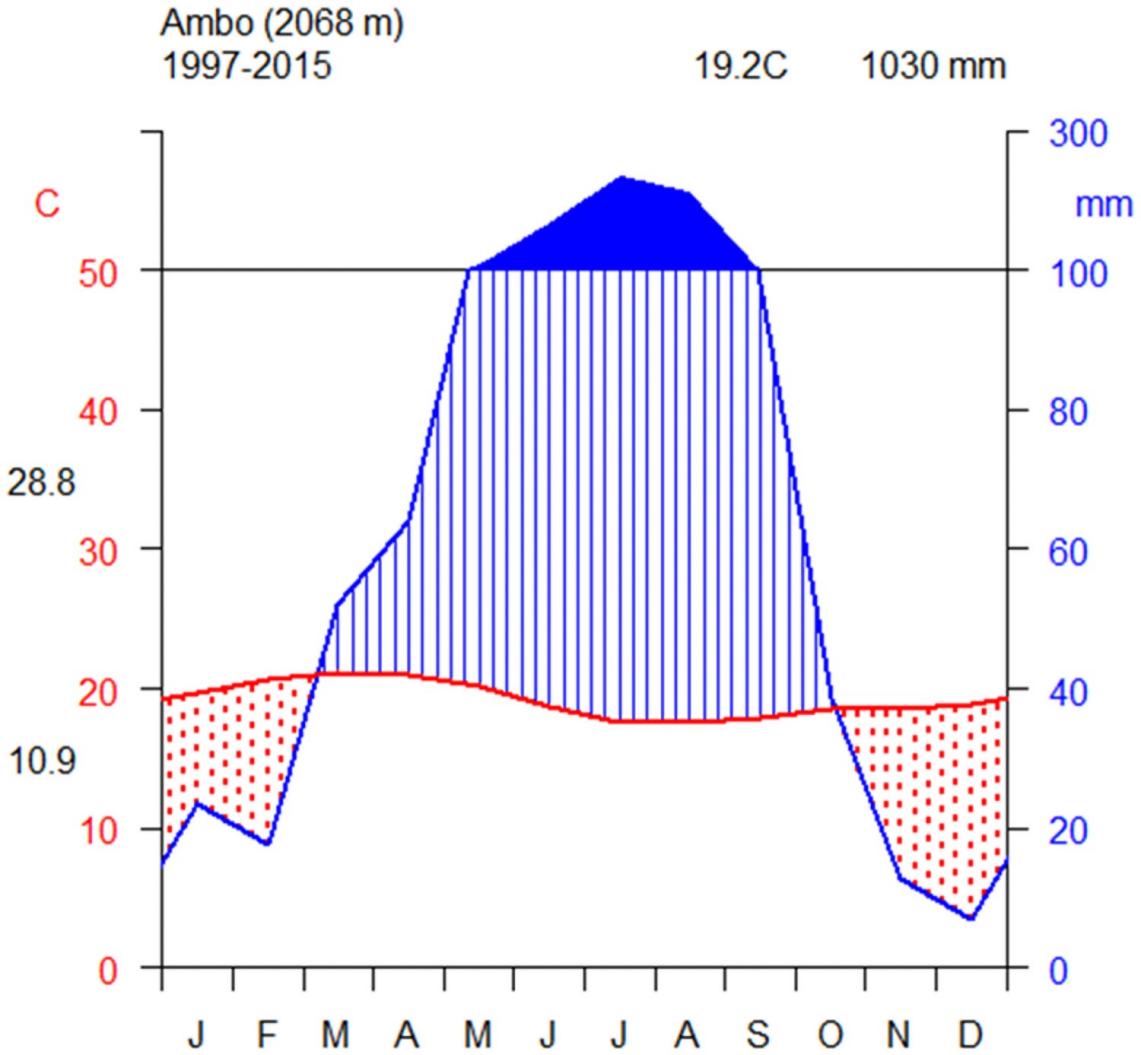
Climadiagram of Ambo weather station near Kibate Forest. The y-axis shows the average annual precipitation (mm) and average annual maximum and minimum temperature (°C) and elevation (meters above sea level), and the x-axis shows the month of the year beginning from January to December.

The primary climate data of the nearest town Ambo were collected from the National Meteorology Agency of Ethiopia. The climadiagram in Fig 2 shows that the study area has a uni-modal rainfall distribution with 1030mm of average annual precipitation from 1997 to 2015. The long rainy season occurs from March to September with a peak in July. The average annual temperature was 19.2°C with 10.9°C and 28.8°C minimum and maximum monthly average temperature, respectively.

### Data collection methods

#### Reconnaissance survey and study site design

The reconnaissance survey of the study area was carried out in Kibate Forest from December 2017 to January 2018. Kibate Forest is located around Wonchi Crater Lake in Southwest Shewa Zone of Oromia Regional State, Ethiopia with the geographical coordinates of 8.775 to 8.793°N and 37.865 to 37.905°E (Fig 1). Specific permission was not required to conduct this study that did not involve the extraction of endangered species. However, a permit was obtained from the Wonchi District Agricultural and Rural Development Office for plant specimen collections in Kibate Forest based on the supporting letter of Addis Ababa University.

For C-stock analysis, the study site was systematically designed to include different altitudinal ranges, slopes, vegetation composition, and anthropogenic factors following Pearson et al. [51] and Condit [52]. Following the reconnaissance survey, five transects were laid out along the three streams, the edge of Wonchi Crater Lake, and along the road track inside the forest. The sample collection was begun at the foot of the forest up to the end of each transect in the hill slope to cover both anthropogenic and physical environmental factors. Using ArcGIS Version 10.5 and GARMIN GPS 72, the altitudinal ranges were partitioned into three classes with an elevation of 100 m a.s.l. interval. All the details of the three classes of elevations and slopes have been described in the previously published paper that was done by the same authors [49].

#### Vegetation and environmental data collection in Kibate Forest

All live vascular plant species within the main plots and subplots were recorded with geographic coordinates using GPS and local and scientific names in the field whenever possible. The plant specimens were collected, pressed, and brought to the National Herbarium of Ethiopia (ETH) with preliminary information. Further determination of the species was conducted using taxonomic keys of different volumes of Flora of Ethiopia and Eritrea. Finally, voucher specimens were deposited at the ETH, Addis Ababa University.

The vegetation composition in Kibate Forest varied with a dominant canopy cover of plant species along the altitudinal gradients. Sixty-six plots of 30 × 30 m (900 m^2^) were therefore established along the five transects with 100 m intervals to include all canopy cover types. Five subplots of 1 × 1 m (four at the corners and one at the center) were nested within each main plot for the sampling of herbaceous species because herbaceous species appear to be related to TC stock. Trees on the border were considered whenever more than 50% of their crown cover falls within the main plot (900 m^2^). Trees with their trunks inside the sampling plot and branches outside were included. For trees of unusual shape, a standard forestry practice was adopted following MacDicken [53]. Accordingly, when the tree branched at breast height or below, the diameter was measured separately for the branches and averaged, whereas, for buttressed tree trunks, diameter measurements were performed to the point just above the buttress. When conditions such as difficult topography and crown structure did not allow using instruments, we reverted to visual estimation to measure the height of tree species. The recommended size plot for measuring biomass was adopted from the previous works [8, 43, 44, 51]. The trunk DBH at 1.3 m height from the ground and height of individual trees with DBH ≥ 2.5 cm and a height of ≥ 2.5 m were measured at each sampling plot using diameter tape and clinometer, starting from the edge and working inward, and marking each woody species to avoid repetition. The scaling factor (SF) was used to convert the field measurements of the AGB tree to ha following Walker et al. [54]. This scaling factor converts the area units from m^2^ to ha (Eq 1):

$$SF=\frac{10,000}{NA}$$ (1)

where SF = scaling factor to convert to ha, 10,000 = meters squared in one ha, and NA = the square area of the main plot (900 m^2^). The biomass estimate is converted to Mg C.

Both physical and anthropogenic factors such as altitude, slope, aspect, human disturbance, and overgrazing by livestock were considered in the sampling procedure. The distributions of AGC and BGC stocks along the three classes of altitude gradients (about 2800–3100 m a.s.l.) were analyzed. Environmental disturbances such as overgrazing by livestock and human disturbances (cutting trees for firewood, charcoal production, and burning of the vegetation for expansion of agricultural land) were noted. The status of negative human interference was coded using the etic approach following Hadera [55], and 0–3 subjective scales are designated as 0 = nil; 1 = low; 2 = moderate; and 3 = heavy. Likewise, livestock grazing intensity was estimated following Woldu and Backeus [56] as 0 = nil; 1 = slight; 2 = moderate; 3 = heavy, and 4 = destructive.

### Data analysis

The data analyses were conducted using R 4.00 following Crawley [57] and Woldu [58]. The biomass analysis was conducted using an allometric equation, which is a nondestructive method. The TC pool was taken as a response variable to fit with some predictor environmental variables (altitude, slope, human intrusion, and overgrazing by livestock) using multiple linear regression and correlation for species richness. The height of the tree (m) was calculated using clinometer (percent scale) as shown in Eq 2.


$$Total\;height=\frac{top\;measurement-bottom\;measurement}{100}\times Distance$$ (2)

The diameter at breast height (cm) was calculated as

$$DBH=\frac{C}{\pi }$$ (3)

where C is circumference, and π = 3.14. Woody species with DBH ≥ 2.5cm and height ≥ 2.5m were used for the analysis of stem density and basal area (BA). The stem density of tree species is expressed as the number of individual stems present per ha of an area. The basal area of the vegetation was computed (Eq 4).


$$BA=\frac{{\pi \times \left(DBH\right)}^{2}}{4}\;or\;0.785\times {\left(DBH\right)}^{2}$$ (4)

For estimation of TC stocks, both AGB and BGB were computed. The allometric equations have been developed from standard C inventory principles and are widely applicable in tropical forests with three forest types (dry, moist, and wet). The AGB of Kibate Forest was estimated (Eq 5) using the allometric equation that was designed for a dry tropical forest type following the improved allometric equation of Chave et al. [6].

$$AGB=0.0673\times ({DBH}^{2}H\rho {)}^{0.9762}$$ (5)

where AGB (kg) is aboveground biomass, DBH is the diameter at breast height in cm, H is total tree height in m, and ρ is wood density for tree species, acquired from the Global Wood Density Database [59] and we used the arithmetic mean for a tropical African forest (0.60 g cm^−3^) for species that were not found in the database following Chave et al. [32]. The C stock of the tropical dry forest is a fraction of 50% of biomass [2, 23, 40, 53, 60]. Thus, aboveground carbon (AGC) was calculated as a conversion factor of 0.5 multiplied by AGB (Eq 6).


AGC=AGB×0.5 (6)

The amount of CO_2_ sequestered in the AGB was estimated using the formula in Eq 7.


$${CO}_{2}\;sequestered=AGC\times 3.67$$ (7)

The standard method for estimation of BGB was obtained as 20% of AGB following MacDicken [53] and IPCC [2], using a synthesis of global data and a conservative ratio shoot- to-root biomass of 5:1 (Eq 8). This conversion factor is widely used in several studies of similar forest types elsewhere in Ethiopia [45–48].


BGB=AGB×0.20 (8)

The estimation of C content and amount of CO_2_ in BGB is the same as that of AGB.

TC stocks were calculated by summing up the C stocks of AGB and BGB following the formula of Pearson et al. [51]. The TC stock for both pools (Mg ha^−1^) of a study area is given by Eq 9.


TCStock=AGC+BGC (9)

## Results

### Taxonomic diversity, height, and DBH of tree species distribution in Kibate Forest

A total of 125 species belonging to 104 genera and 52 families were identified in Kibate Forest. All taxonomic data including endemic species, genus, family, habit, author, and local names of species are presented separately in S1 Table. The highest number of species (54%) was herbs, followed by shrub (24%) and tree (16%) species in the study area. The total stem density of 20 tree species with a height of ≥ 2.5 m accounted for 98.7 stems ha^−1^. Four species (*Erica arborea*, *Myrica salicifolia*, *Olea europaea* subsp. *cuspidata* and *Olinia rochetiana*) contributed to more than half (57.8%) of the total stem density. Nine woody species including *Juniperus procera*, *Nuxia congesta*, *Ilex mitis* var. *mitis*, *Olea europaea* subsp. *cuspidata*, *Myrica salicifolia*, *Agarista salicifolia*, *Hagenia abyssinica*, *Buddleja polystachya*, and *Myrsine melanophloeos* accounted for the highest proportion (74%) of BA. The average DBH and height of tree species in the study area were 30.14 ± 6.27 cm and 16.3 ± 4.30 m, respectively (Table 1).

**Table 1:** Summary of stem density, basal area, average diameter at breast height, and height of 20 tree species in Kibate Forest.

Name of Species	Number of stems	Average DBH (cm)	Average height (m)	Stem density (No. ha^−1^)	BA (%)
***Agarista salicifolia***	136	33.49	7.04	3.86	5.1
***Brucea antidysenterica***	2	10.19	8.5	0.05	0.5
***Buddleja polystachya***	16	29.88	10.5	0.45	4.0
***Erica arborea***	532	19.59	6.5	15.08	1.7
***Galiniera saxifraga***	34	20.60	8.5	0.96	1.9
***Hagenia abyssinica***	266	32.65	18.5	7.54	4.8
***Halleria lucida***	18	20.06	10.5	0.51	1.8
***Hypericum revolutum***	59	9.83	10.5	1.67	0.4
***Ilex mitis* var. *mitis***	191	44.60	38.5	5.42	9.0
***Juniperus procera***	177	64.02	38.5	5.02	18.6
***Maesa lanceolata***	15	26.01	18.5	0.42	3.1
***Maytenus addat***	21	24.61	28.5	0.59	2.7
***Myrica salicifolia***	418	38.79	13.5	11.85	6.8
***Myrsine melanophloeos***	170	27.92	18.5	4.81	3.5
***Nuxia congesta***	249	60.83	13.5	7.05	16.8
***Olea europaea* subsp. *cuspidata***	557	43.17	13.5	15.79	8.4
***Olinia rochetiana***	506	22.71	14.5	14.34	2.3
***Pittosporum viridiflorum***	6	26.4	13.5	0.17	3.2
***Protea gaguedi***	101	25.16	6.5	2.86	2.9
***Schefflera volkensii***	8	22.34	28.5	0.22	2.3

BA = Basal area; DBH = Diameter at breast height.

#### Relationships between TC stocks and species richness

**Fig 3:**
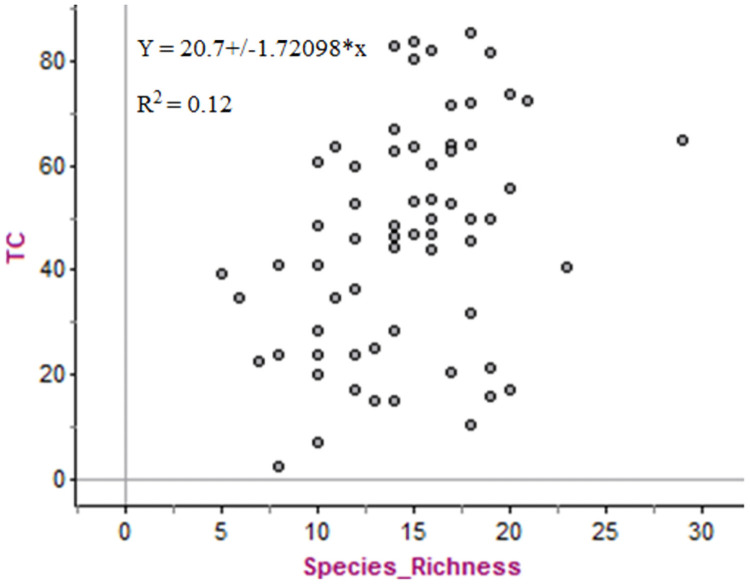
Scatter plot showing the correlation between TC stocks and species richness in Kibate Forest.

There was a weak positive correlation between TC stock and species richness (r = 0.34). In Fig 3, the scatter plot shows that 12% of the total variation in TC stock is explained by the relationship between species richness and TC stocks, which indicates the limited distribution of large-size tree species along the transects in Kibate Forest.

### Carbon stocks in the upper canopy of woody species in Kibate Forest

Most of the biomass and C stock were found within the large trees. Twenty tree species with DBH ≥ 2.5 cm and height ≥ 2.5 m were therefore considered when estimating TC stocks. The highest C stocks (67.4%) were recorded in *Juniperus procera* with a sum of AGC and BGC of 35.3 (Mg ha^−1^), followed by *Ilex mitis* var. *mitis* (17.0 Mg ha^−1^), *Nuxia congesta* (11.5 Mg ha^−1^), and *Olea europaea* subsp. *cuspidata* (5.5 Mg ha^−1^). This indicates that on average, *Juniperus procera* sequestered the highest amount of CO_2_ per tree. The mean TC stocks of the 20 tree species accounted for 5.14 ± 1.83 Mg ha^−1^, of which 4.28 ± 1.52 Mg ha^−1^ were AGC and 0.86 ± 0.30 Mg ha^−1^ were BGC (Table 2).

**Table 2:** Carbon stock pools (Mg ha^−1^) across the tree species in Kibate Forest.

*Species*	AGB	AGC	CO_2_ in AGB	BGB	BGC	CO_2_ in BGB	TB	TC Stock
***Agarista salicifolia***	2.94	1.47	5.39	0.58	0.29	1.06	3.54	1.77
***Brucea antidysenterica***	0.32	0.16	0.59	0.06	0.03	0.11	0.4	0.20
***Buddleja polystachya***	3.56	1.78	6.53	0.72	0.36	1.32	4.28	2.14
***Erica arborea***	0.9	0.45	1.65	0.18	0.09	0.33	1.08	0.54
***Galiniera saxifraga***	1.34	0.67	2.46	0.26	0.13	0.48	1.62	0.81
***Hagenia abyssinica***	7.46	3.73	13.69	1.5	0.75	2.75	8.96	4.48
***Halleria lucida***	1.52	0.76	2.79	0.3	0.15	0.55	1.84	0.92
***Hypericum revolutum***	0.38	0.19	0.70	0.08	0.04	0.15	0.46	0.23
***Ilex mitis* var. *mitis***	28.4	14.20	52.11	5.68	2.84	10.42	34.06	17.03
***Juniperus procera***	58.8	29.40	107.90	11.76	5.88	21.58	70.56	35.28
***Maesa lanceolata***	4.72	2.36	8.66	0.94	0.47	1.72	5.66	2.83
***Maytenus addat***	6.5	3.25	11.93	1.3	0.65	2.39	7.8	3.90
***Myrica salicifolia***	7.56	3.78	13.87	1.52	0.76	2.79	9.08	4.54
***Myrsine melanophloeos***	5.34	2.67	9.80	1.06	0.53	1.95	6.42	3.21
***Nuxia congesta***	19.16	9.58	35.16	3.84	1.92	7.05	23	11.50
***Olea europaea* subsp. *Cuspidata***	9.1	4.55	16.70	1.82	0.91	3.34	10.92	5.46
***Olinia rochetiana***	2.8	1.40	5.14	0.56	0.28	1.03	3.36	1.68
***Pittosporum viridiflorum***	3.42	1.71	6.28	0.68	0.34	1.25	4.12	2.06
***Protea gaguedi***	1.6	0.80	2.94	0.32	0.16	0.59	1.92	0.96
***Schefflera volkensii***	5.32	2.66	9.76	1.06	0.53	1.95	6.38	3.19
**Mean**	8.56±3.05	4.28±1.52	15.70±5.59	1.71±0.61	0.86±0.30	3.14±1.12	10.27±3.66	5.14±1.83
**Total**	171.14	85.60	314.15	34.22	17.11	62.79	205.44	102.72

AGB = Aboveground biomass, AGC = Aboveground carbon, BGB = Belowground biomass, BGC = Belowground carbon, TB = Total biomass, and TC = Total carbon.

Both above- and belowground C pools and anthropogenic impacts across all plots are presented separately in S2 Table. The result showed that the total average of tree biomass was 91.9 ± 10.01 Mg ha^−1^, ranging from 4.6 to 171.0 Mg ha^−1^, where tree species with small and large DBH and height, respectively, were dominating. The great variation in tree biomass resulted in high variation between plots in C stocks ranging from 2.3 to 85.5 Mg ha^−1^. Kibate Forest had a TC stock of 3031 Mg ha^−1^ with a mean TC stock of 45.9 ± 5.17 Mg ha^−1^. The mean AGC for all plots was 38.3 ± 4.31 Mg ha^−1^. Of the TC pools, about 504 Mg ha^−1^ was BGC, and 2526 Mg ha^−1^ was AGC. The overall values of biomass and C stock (Mg ha^−1^) of the study site are shown in Table 3.

**Table 3:** Summary of total and mean values of biomass and C stock (Mg ha^−1^) in above- and belowground compartments in Kibate Forest.

	AGB	BGB	TB	AGC	BGC	TC Stock
**Mean**	76.6±8.62	15.3±1.82	91.9±10.01	38.3±4.31	7.7±0.91	45.9±5.17
**Total**	5053	1009	6062	2526.46	505	3031

AGB = Aboveground biomass, AGC = Aboveground carbon, BGB = Belowground biomass, BGC = Belowground carbon, TB = Total biomass, and TC = Total carbon.

### The relationship of environmental variables to carbon stock distribution in Kibate Forest

The results showed that the highest mean C stock with 55.6 ± 3.71 Mg ha^−1^ (a total of 945.7 Mg ha^−1^), of which 46.3 ± 3.20 (786.2) Mg ha^−1^ is in AGC and 9.4 ± 0.78 (159.5) Mg ha^−1^ is in BGC, was recorded in the lower altitude class ranging from 2800 to 2900 m a.s.l. The middle altitude class (2901–3000 m a.s.l.) accounted for 45.9 ± 3.77 Mg ha^−1^ with 38.3 ± 3.12 (AGC) and 7.7 ± 0.64 (BGC) Mg ha^−1^ (Table 4).

**Table 4:** Mean TC stocks (Mg ha^−1^) in above- and belowground C pools along three altitudinal gradients.

Altitude class	Altitude range (m a.s.l.)	No. of plots	AGC	BGC	TC Stock
**Lower**	2800–2900	17	46.3±3.20	9.4±0.78	55.6±3.71
**Middle**	2901–3000	37	38.3±3.12	7.6±0.64	45.9±3.77
**Upper**	≧ 3001	12	26.8±4.16	5.4±0.87	32.2±4.99

AGC = Aboveground carbon, BGC = Belowground carbon, and TC = Total carbon.

The results in Table 5 revealed no significant reduction in TC stock with increasing altitude (P = 0.74) and slope (P = 0.95). On the other hand, livestock grazing and human intrusions affected the distribution of TC stocks negatively and significantly (P < 0.001) in Kibate Forest.

**Table 5:** Predictive physical environmental and anthropogenic factors for the response of TC stock distributions in Kibate Forest.

	Estimate (C stocks in Mg ha^−1^)	Std. Error	t value	Pr(≥|t|)	Sig.
**Intercept**	106.786	82.124	1.300	0.198	
**Altitude**	−0.010	0.0298	−0.337	0.737	
**HI**	−12.392	0.770	−16.084	<2e−11	***
**LG**	−9.314	0.737	−12.641	<2e−07	***
**Slope**	−0.007	0.127	0.059	0.953	

Altitude (m a.s.l.); HI = Human impacts; LG = Livestock grazing; Slope (%); Residual standard error: 5.638 on 61 degrees of freedom; Multiple R-squared: 0.9349, Adjusted R-squared: 0.9307; F-statistic: 219.1 on 4 and 61 degrees of freedom, P-value: < 2.2e−16.

## Discussion

As has been done for forest cover which is subjected to deforestation and degradation [44], the biomass of tree species of Kibate Forest was calculated using an allometric equation following the method of Chave et al. [6]. The C stock estimation can be computed either by using a destructive method of biomass estimation or by using a conversion factor that in turn varies depending on the plant growth habit, forest type, and method [23, 41, 60]. According to Gao et al. [41], the acceptable conversion factors include either the C concentration of 50% for both woody and non–woody species tissues or 50% for woody and 45% for non–woody species tissues. Therefore, the C stocks of a tree are computed as the tree biomass predictions multiplied by C conversion factors, either as an acceptable common constant (i.e., 0.5) [2, 41] or an empirical constant based on available data [60]. In the present study, the C stocks of the tree were calculated using the acceptable and default method of global carbon factor 0.5, as suggested in IPCC guidelines [2, 23, 44, 48, 53, 60]. In Kibate Forest, C stock in the plots sampled was not uniform, and the variation ranged from 14.9 (plot 44) to 85.5 Mg ha^−1^ (plot 8). However, TC at three plots accounting for ~5% of the total area was considerably smaller (plot 11 = 2.31; plot 41 = 6.8 and plot 43 = 10.4 Mg ha^−1^). The substantial variations of C stocks between plots could be due to wide variations of the distribution of large trunk tree species. Hence, sites with higher C stocks could be due to more abundant large diameter and tall trees whereas those sites with the least C stocks could be due to relatively high human and livestock disturbances as discussed by Srinivas and Sundarapandian [61]. The mean TC stock of all the plots (45.9 ± 5.17 Mg ha^−1^) of the present study was found within the global range value (14–123 Mg C ha^−1^) as reported by Murphy and Lugo [62] as well as within the range of the mean C stock value (39–334 Mg ha^−1^) of the dry tropical forests as reported by Becknell et al. [63]. However, the mean AGB (76.6 ± 8.62 Mg ha^−1^) of Kibate Forest is lower than that of 260 African tropical forests (395.7 Mg ha^−1^) estimated by Lewis et al. [64].

In a wider perspective, the dry evergreen montane forest ecosystem is severely threatened due to human and livestock pressures, which resulted in the loss of forest cover and biodiversity [25, 50, 61, 65]. The results of the present study in Table 5 confirmed that human impacts, including the expansion of agricultural land and cutting tall trees as well as overgrazing by livestock, had negatively and significantly affected TC stock distribution. Brown and Lugo [66] showed that accumulated biomass in forest ecosystems may vary with variation in the physical environment (altitude and slope), climate (precipitation and temperature), and intensity of anthropogenic disturbances (both by humans and livestock). Furthermore, the felling of tall trees for various purposes could be one of the reasons for the preponderance of herbaceous species over woody species in the study area. The reduction of biomass mainly because of deforestation and reduction in the forest cover is a global concern [8–11]. It is reported that deforestation and land-use changes with increasing population will contribute to nearly one-third of anthropogenic CO_2_ emissions [18, 67]. In Sub-Saharan African countries including Ethiopia, the dry tropical forest ecosystem is a serious issue concerning the loss of forest cover and biodiversity due to impediments of high anthropogenic pressures [1, 20–22, 61, 66].

It is important to understand how tree species richness influences TC stocks [7]. Many researchers agreed that large trunk trees can sequester large amounts of C per tree due to the direct relationship between biomass and C stock [47, 48, 64]. In Kibate Forest, the highest C stocks were recorded in six large trees (Table 2), which are also the dominant and characteristic species in a dry evergreen montane forest [48]. Species that stored the highest C stock were the dominant species showing higher BA as reported by Dibaba et al. [47] for a study conducted in Gerba-Dima Forest in southwestern Ethiopia. Tree species represented by individuals with larger DBH and height have a significant contribution to C storage as shown in Table 1. Liu et al. [7] indicated that sites with high species richness had higher C stocks than sites with low richness even though they were rich in herbaceous species. A weak correlation between TC stock and species richness as shown in Kibate Forest (Fig 3) confirmed that not only the total number of species but also the distribution of large trunk tree species significantly determine the C stock potential. Thus, an ecosystem that is subjected to anthropogenic intervention needs more attention to restore and conserve the remaining forests that harbor large trunk tree species [8–11, 65].

In addition to anthropogenic factors, physical environmental factors such as altitude and slope have both positive and negative influences on the C stock distribution of different forests in Ethiopia [38, 47, 48]. In line with the present study (Table 4), the highest C stock was recorded in the lower altitude class, followed by the middle and upper classes of forest C stocks in the lowland area of Semien Mountains [46]. However, there was no significant relationship between TC stocks and altitude in Kibate Forest. This could be due to the short-range (2800–3100 m a.s.l.) of altitude classes in Kibate Forest as similar findings were obtained in dry Afromontane forests of Awi Zone [48] and the availability of tree species [68, 69]. The effect of elevation was also associated with temperature, precipitation, and edaphic changes [38]. Hence, further research work on these associated factors with C stocks is important to reach a definitive conclusion.

Despite rapid depletion of forests, Ethiopia has considerable forest resources in the Horn of Africa [13, 14, 19]. However, the gradual reduction of forest cover has led to a decrease of C stocks of forest biomass and soil degradation beyond restoration. Thus, understanding the balance between the C input rates of biomass and decomposition rates of soil organic matter is crucial for forest soil management practices [18, 67, 70]. Hence, serious formal action is needed to curb anthropogenic pressures such as the expansion of agricultural land in the peripheral forest areas, felling of trees for various purposes, and overgrazing by livestock, and to take conservation measures in the dry evergreen forest to enhance C sink in the climate change scenario. The protection and sustainable management of forest C stocks need to be given serious attention to promote REDD (reducing emissions from deforestation and forest degradation) and REDD+ projects and thereby make mitigation of climate change and conservation of biodiversity [1, 12, 25, 46, 67, 71, 72]. Thus, for a forest that is rich in endemic species and taxonomic diversity with considerable C stock, conservation of biodiversity and market-based C stock potential assessment need to be aligned.

## Conclusions

A total of 125 species were recorded in Kibate Forest, out of which 20 species were tree forms. Four species with large-sized individual trees contributed to more than half of the total stem density (57.8%). Eleven woody species (*J*. *procera*, *N*. *congesta*, *I*. *mitis* var. *mitis*, *O*. *europaea* subsp. *cuspidata*, *M*. *salicifolia*, *A*. *salicifolia*, *H*. *abyssinica*, *B*. *polystachya*, *M*. *melanophloeos*, *E*. *arborea*, and *O*. *rochetiana*) contributed the highest to BA, stem density and C stocks in the study area. The result showed that C-stock distribution varied between plots related to the abundance of large trunk tree distribution mainly due to anthropogenic pressures as also shown by Solomon et al. [23]. Anthropogenic factors, particularly the expansion of agricultural land, felling of trees, and overgrazing by livestock, significantly affected the C stocks in Kibate Forest. Our findings showed that Kibate Forest makes a significant contribution to C sequestration in the region and can generate C credits for Ethiopia. However, three plots in the study area accounting for ~5% of the total area were considerably smaller than the minimum global range value of 14 Mg ha^−1^ and need special attention to prevent further degradation and to maintain sustainable use aiming at improving the C stock to the global range.

## Supporting information

S1 Table Inventory of plant species with respective local name, family, habit, and location in Kibate Forest. H = Habit form; Habit (F = Fern, H = Herb, L = Liana, T = Tree, S = Shrub). + for endemic species and ++ for near-endemic species that are found only in Ethiopia and Eritrea. Missing plot numbers are those which have no new species encountered other than species that have already been recorded in the preceding plots.
(PDF)

S2 Table Environmental variables, tree biomass, and C stock (Mg ha^−1^) within 66 plots in Kibate Forest. AGB = Aboveground biomass, BGB = Belowground biomass, TB = Total biomass, AGC = Aboveground carbon stock, BGC = Belowground carbon stock, TC = Total carbon stock, HI = Human impact, and LG = Livestock grazing.
(PDF)
