# Supplementary material for: Carbon stocks of above- and belowground tree biomass in Kibate Forest around Wonchi Crater Lake, Central Highland of Ethiopia
Source: PLoS One. 2021 Jul 9;16(7):e0254231. doi: 10.1371/journal.pone.0254231 (PMC8270183; doi:10.1371/journal.pone.0254231)
Supplement: S1 Table — H = Habit form; Habit (F = Fern, H = Herb, L = Liana, T = Tree, S = Shrub). + for endemic species and ++ for near-endemic species that are found only in Ethiopia and Eritrea. Missing plot numbers are those which have no new species encountered other than species that have already been recorded in the preceding plots. (PDF) [file pone.0254231.s001.pdf]

S1 Table. Inventory of plant species with respective local name, family, habit and location in Kibate Forest. H = Habit form; Habit (F = Fern, H = Herb, L = Liana, T = Tree, S = Shrub). + for endemic species and ++ for near endemic species that are found only in Ethiopia and Eritrea. Missing plot numbers are those which have no new species encountered other than species which have already been recorded in the preceding plots.

No.	**Scientific name**	**Local name (Oromic/Amharic)**	**Family**	**Ha**	**Geographical location (based on GPS record)**	**Plot No.**	**Collection No.**
1	*Achyranthes aspera* L.	Samaa/ Telenj	Amaranthaceae	H	2973 m; 08^{0}46.028'; 037^{0}53.350'	8	MM570
2	*Achyrospermum schimperi* (Hochst. ex Briq.) Perkins	Qorcha Lega/ Balandalecha	Lamiaceae	H	2839 m; 08^{0}46.718'; 037^{0}53.967'	55	MM542
3	*Adenocarpus mannii* (Hook. j) Hook. f.	Tosign Mesay	Fabaceae	S	3015 m; 08^{0}47.203'; 037^{0}52.919'	30	MM539
4	*Adiantum hispidulum* Sw.	Rough Maidenhair (Eng)	Adiantaceae	F	2842 m; 08^{0}46.809'; 037^{0}53.044'	52	MM566
5	*Adiantum poiretii* Wikstr.	Rosy Maidenhair (Eng)	Adiantaceae	F	2964 m; 08^{0}46.790'; 037^{0}53.065'	18	MM505
6	*Agarista salicifolia* (Comm. ex Lam.) Don.	Erepisa/Sootirii	Ericaceae	T	2876 m; 08^{0}47.097'; 037^{0}54.022'	1	MM469
7	*Alchemilla abyssinica* Fresen.	Yeayit Joro	Rosaceae	H	2964 m; 08^{0}47.014'; 037^{0}53.240'	17	MM501
8	*Andoropogon amethystinus* Steud.	Balaami/keysar	Poaceae	H	2924 m; 08^{0}47.088'; 037^{0}52.956'	27	MM535
9	*Argyrolobium ramosissimum* Bak.	Soogidda haree/Gerengere	Fabaceae	H	2876 m; 08^{0}47.097'; 037^{0}54.022'	1	MM468
10	*Arundinaria alpina* K. Schum.	Shimella/Kerkeha	Poaceae	S	2856 m; 08^{0}46.704'; 037^{0}53.000'	31	MM545
11	*Arundo donax* L.	Shenbeqo	Poaceae	S	2913 m; 08^{0}46.584'; 037^{0}52.878'	56	MM571
12	*Asparagus africanus* Lam.	Seertii/Qestanicha	Asparagaceae	S	2953 m; 08^{0}46.964'; 037^{0}53.813'	3	MM479
13	*Astragalus atropilosulus* (Hoehst.) Bunge	Guaya Mesay	Fabaceae	H	2842 m; 08^{0}46.809'; 037^{0}53.044'	52	MM573

14	*Bartsia longiflora* Hochst. ex Benth.	Selit Mesay	Scrophulariaceae	H	3015 m; 08^{0}47.203'; 037^{0}52.919'	30	MM485
15	*Bidens pachyloma*[+] (Oliv. & Hiern) Cufod.	Yemeskel Abeba	Asteraceae	H	3073 m; 08^{0}46.928'; 037^{0}52.558'	48	MM580
16	*Brucea antidysenterica* J. F. Mill.	Qomengo /Waginos	Simaroubaceae	T	2956 m; 08^{0}46.584'; 037^{0}52.878'	38	MM520
17	*Buddleja polystachya* Fresen.	Amfara/Anfar	Loganiaceae	T	2958 m; 08^{0}46.948'; 037^{0}53.738'	4	MM480
18	*Campanula edulis* Forssk.	Bell shaped flower (Eng)	Campanulaceae	H	3040 m; 08^{0}46.834'; 037^{0}52.765'	24	MM514
19	*Carduus nyassanus* (S. Moore) R.E. Fr.	Balao Woranti	Asteraceae	H	2964 m; 08^{0}46.790'; 037^{0}53.065'	18	MM506
20	*Carex acutiformis* Ehrb.	Gicha/ashenda	Cyperaceae	H	2811 m; 08^{0}46.677'; 037^{0}53.154'	51	MM551
21	*Carex steudneri* Bock		Cyperaceae	H	2811 m; 08^{0}46.677'; 037^{0}53.154'	51	MM558
22	*Cichorium intybus* L.	Chicory	Asteraceae	H	2842 m; 08^{0}46.809'; 037^{0}53.044'	53	MM576
23	*Cineraria abyssinica* Sch. Bip. ex A. Rich.	Abeba Meskele	Asteraceae	H	2953 m; 08^{0}46.964'; 037^{0}53.813'	3	MM478
24	*Conyza hypoleuca* A. Rich.	Haxxawi/Nechillo	Asteraceae	S	2973 m; 08^{0}46.963'; 037^{0}53.638'	5	MM484
25	*Conyza steudelii* Sch. Bip. ex A. Rich.	Yeyfat Kitel/Entesa	Asteraceae	H	2944 m; 08^{0}46.632'; 037^{0}52.783'	17	MM562
26	*Crepis rueppellii* Sch. Bip.	Anannoo/Yefyelwetet	Asteraceae	H	2973 m; 08^{0}46.963'; 037^{0}53.638'	5	MM482
27	*Crepis tenerrima* (Sch. Bip. ex A. Rich.) R. E. Fries	Demastefi	Asteraceae	H	2842 m; 08^{0}46.809'; 037^{0}53.044'	53	MM574
28	*Crotalaria* sp.		Fabaceae	S	2842 m; 08^{0}46.809'; 037^{0}53.044'	53	MM578
29	*Cyathula cylindrica* Moq.	Tlashet	Amaranthaceae	H	2973 m; 08^{0}46.028'; 037^{0}53.350'	8	MM487
30	*Cynoglossum amplifolium* Hochst. ex A.DC.in DC.	Chogogitii/Yeshiwuta	Boraginaceae	H	2964 m; 08^{0}46.790'; 037^{0}53.065'	18	MM502
31	*Discopodium penninervium* Hochst.	Chochinga/Qelaho	Solanaceae	S	2964 m; 08^{0}47.014'; 037^{0}53.240'	17	MM496
32	*Distictis buccinatonia* (DC.) A. Gentry	Echilibe mesay	Bignoniaceae	S	2866 m; 08^{0}46.912'; 037^{0}53.023'	54	MM543
33	*Droguetia iners* (Forssk.) Schweinf	Kechinu	Urticaceae	H	2866 m; 08^{0}46.912'; 037^{0}53.023'	54	MM522
34	*Echinops giganteus* A. Rich.	Mata-boqe	Asteraceae	H	2973 m; 08^{0}46.028'; 037^{0}53.350'	8	MM486

35	*Echinops macrochaetus* Fresen.	Koshesshilla	Asteraceae	S	3015 m; $08^0$47.203'; $037^0$52.919'	30	MM541
36	*Emilia serpentinus*$^+$ Mesfin & Beentje		Asteraceae	H	2902 m; $08^0$46.790'; $037^0$52.972'	20	MM507
37	*Englerina woodfordioides* (Schweinf) M Gilbert	Digelu/Teketiya	Loranthaceae	E	2981 m; $08^0$47.171'; $037^0$53.177'	11	MM490
38	*Epipactis africana* Rendle	Yelamtut	Orchidaceae	H	3016 m; $08^0$46.831'; $037^0$52.776'	24	MM577
39	*Erica arborea* L.	Kemete/Asta	Ericaceae	T	2876 m; $08^0$47.097'; $037^0$54.022'	1	MM457
40	*Euphoria platyphyllos* L.	Anano/Anterifa	Euphorbiaceae	H	3073 m; $08^0$46.928'; $037^0$52.558'	66	MM565
41	*Festuca simensis* Hochst. ex A. Rich.	Garbu	Poaceae	H	2811 m; $08^0$46.677'; $037^0$53.154'	51	MM559
42	*Galiniera saxifraga* (Hochst.) Bridson	Mukadamo/Buna Mesay	Rubiaceae	T	2973 m; $08^0$46.963'; $037^0$53.638'	5	MM483
43	*Galium simense* Fresen.	Ashekit	Rubiaceae	H	2934 m; $08^0$46.678'; $037^0$52.962'	5	MM556
44	*Girardinia bullosa* (Steudel) Wedd.	Dobbi	Urticaceae	H	2839 m; $08^0$46.718'; $037^0$53967'	52	MM538
45	*Gnaphalium rubriflorum* Hilliard	Nibweda Mesay	Asteraceae	H	2896 m; $08^0$46.942'; $037^0$53.016'	25	MM519
46	*Hagenia abyssinica* (Bruce) J.F.Gmelin	Hexxoo/Kosso	Rosaceae	T	3015 m; $08^0$47.203'; $037^0$52.919'	30	MM540
47	*Halleria lucida* L.	Echilibe/Misnkero	Scrophulariaceae	T	2981 m; $08^0$46.845'; $037^0$52.801'	23	MM509
48	*Haplocarpha schimperi* (Sch.-Bip.) Beauv.	Getin	Asteraceae	H	2842 m; $08^0$46.809'; $037^0$53.044'	53	MM525
49	*Helichrysum formosissimum* Sch.Bip.ex A.Rich.	Everlasting Flower (Eng)	Asteraceae	H	2866 m; $08^0$46.614'; $037^0$53.312'	12	MM493
50	*Helichrysum hedbergianum*$^+$Mesfin & Reilly	Necho	Asteraceae	H	2973 m; $08^0$47.104'; $037^0$53.219'	10	MM568
51	*Helichrysum schimperi* (Sch.Bip. ex A. Rich.) Moeser	Baalcii	Asteraceae	H	2988 m; $08^0$46.738'; $037^0$52.768'	42	MM552
52	*Hypericum revolutum* Vahl	Hiniyee/Amja	Hypericaceae	T	2940 m; $08^0$47.067'; $037^0$53.875'	2	MM476
53	*Hypoestes forskaolii* (Vahl) R. Br.	Keymatebiya	Acanthaceae	H	2876 m; $08^0$47.097'; $037^0$54.022'	1	MM463

54	*Hypoestes triflora (Forssk.)* Roem &Schult.	Dergu/Keymatebiya	Acanthaceae	H	2964 m; 08^{0}46.790'; 037^{0}53.065'	18	MM503
55	*Ilex mitis* (L.) Radlk. var. *mitis*	Miesa/daalacho	Aquifoliaceae	T	2940 m; 08^{0}47.067'; 037^{0}53.875'	2	MM475
56	*Inula confertiflora*$^+$ A. Rich.	Soyama/Weynagift	Asteraceae	S	2940 m; 08^{0}47.067'; 037^{0}53.875'	2	MM474
57	*Isodon schimperi* (Vatke) J.K. Morton	Yefiyel Gomen	Lamiaceae	H	2981 m; 08^{0}46.845'; 037^{0}52.801'	23	MM512
58	*Jasminum stans*$^+$ Pax	Intaabuyee/Ano Kitel	Oleaceae	S	2866 m; 08^{0}46.614'; 037^{0}53.312'	12	MM491
59	*Juniperus procera* Hochst. ex. Endl.	Gantira/Yabesha Tid	Cupressaceae	T	2876 m; 08^{0}47.097'; 037^{0}54.022'	1	MM464
60	*Kalanchoe petitiana*$^+$ A. Rich. var. *neumannii*	Bosoqe/Endahula	Crassulaceae	H	2876 m; 08^{0}47.097'; 037^{0}54.022'	1	MM470
61	*Laggera tomentosa*$^+$ (Sch. Bip.ex A. Rich.) Oliv. & Hiern.	Kaskasse/Alashume	Asteraceae	S	2839 m; 08^{0}46.718'; 037^{0}53.967'	55	MM524
62	*Lobelia giberroa* Hemsl.	Maranga/Gibera/Daju	Lobeliaceae	S	2876 m; 08^{0}46.813'; 037^{0}53.244'	14	MM494
63	*Maesa lanceolata* Forssk.	Abbayyii/Qelewa	Myrsinaceae	T	3044 m; 08^{0}47.132'; 037^{0}52.803'	64	MM526
64	*Maytenus addat*$^+$ (Loes.) Sebsebe	Kombolcha/Atatt	Celastraceae	T	3015 m; 08^{0}47.203'; 037^{0}52.919'	30	MM536
65	*Maytenus gracilipes* (Welw. ex Oliv.) Exell	Kombolicha/Atat	Celastraceae	S	2848 m; 08^{0}46.719'; 037^{0}53.072'	52	MM561
66	*Mentha x piperita* L.	Dufa/Shall mesay	Lamiaceae	H	2888 m; 08^{0}47.028'; 037^{0}52.983'	26	MM533
67	*Mikania capensis* DC.	Purple stem	Asteraceae	H	3040 m; 08^{0}46.834'; 037^{0}52.765'	24	MM515
68	*Mikaniopsis clematoides*$^+$ (Sch. Bip. ex A. Rich.) Milne-Redh.	Hadi/Sirutebey	Asteraceae	H	2866 m; 08^{0}46.614'; 037^{0}53.312'	12	MM532
69	*Myrica salicifolia* Hochst. ex A. Rich.	Borodo/Shinet	Myricaceae	T	2876 m; 08^{0}47.097'; 037^{0}54.022'	1	MM461
70	*Myrsine africana* L.	Kachama/Kechemo	Myrsinaceae	S	2876 m; 08^{0}47.097'; 037^{0}54.022'	1	MM460
71	*Myrsine melanophloeos* (L.) R. Br.	Odabeda/Weyil	Myrsinaceae	T	2876 m; 08^{0}47.097'; 037^{0}54.022'	1	MM471
72	*Nuxia congesta* R.Br. ex Fresen.	Qawwisa/Chechiho	Loganiaceae	T	2876 m; 08^{0}47.097'; 037^{0}54.022'	1	MM473
73	*Oldenlandia lancifolia* (Schumach.) DC.	Ashekt mesay	Rubiaceae	H	2981 m; 08^{0}46.845'; 037^{0}52.801'	23	MM511

74	*Olea europaea* L. subsp. *cuspidata*	Ejersa/Weyra	Oleaceae	T	2876 m; 08^{0}47.097'; 037^{0}54.022'	1	MM467
75	*Olinia rochetiana* A. Juss	Soolee/Dalacho	Oliniaceae	T	2876 m; 08^{0}47.097'; 037^{0}54.022'	1	MM466
76	*Orobanche ramosa* L.	Amichodore/Atqurit	Orobanchaceae	H	2956 m; 08^{0}46.584'; 037^{0}52.878'	38	MM527
77	*Osyris quadripartita* Decn.	Wato/Keret	Santalaceae	S	2876 m; 08^{0}47.097'; 037^{0}54.022'	1	MM564
78	*Parochaetus communis* D. Don	Yemdr Koso	Fabaceae	H	2856 m; 08^{0}46.704'; 037^{0}53.000'	31	MM544
79	*Pelargonium wonchiense*$^{+}$ Vorster & M. G. Gilbert		Geraniaceae	H	2913 m; 08^{0}46.584'; 037^{0}52.878'	56	MM530
80	*Pentas schimperiana* (A. Rich.) Vatke subsp. *schimperiana*	Qaasii/Weynagift	Rubiaceae	S	2973 m; 08^{0}46.028'; 037^{0}53.350'	8	MM488
81	*Phytolacca dodecandra* L'He'rit.	Endode/Endod	Phytolaccaceae	S	3061 m; 08^{0}46.837'; 037^{0}52.667'	47	MM534
82	*Pilea johnstonii* Oliv.	Opp.leaf	Urticaceae	H	2934 m; 08^{0}46.678'; 037^{0}52.962'	59	MM555
83	*Pittosporum viridiflorum* Sims.	Elilibaye/Shole	Pittosporaceae	T	2981 m; 08^{0}46.845'; 037^{0}52.801'	23	MM510
84	*Plectocephalus varians*$^{++}$ (A. Rich.) C. Jeffrey ex Cufod.	Etse-Yohannes	Asteraceae	H	3040 m; 08^{0}46.834'; 037^{0}52.765'	24	MM517
85	*Polygala abyssinica* Fres.	Etse-Libona	Polygalaceae	H	3015 m; 08^{0}47.203'; 037^{0}52.919'	30	MM572
86	*Primula verticillata*$^{++}$ Forssk. subsp. *simensis*	Baldega/ayinabra	Primulaceae	H	2964 m; 08^{0}47.014'; 037^{0}53.240'	17	MM499
87	*Protea gaguedi* J. F. Gmel.	Daanishute/Awra	Proteaceae	T	2981 m; 08^{0}46.845'; 037^{0}52.801'	23	MM508
88	*Pteridium aquilinum* (L.) subsp. *capense*	Karo/Brachen	Hypolepidaceae	F	2932 m; 08^{0}46.932'; 037^{0}53.217'	16	MM497
89	*Pteris cretica* L.	Sar mesay	Pteridaceae	F	2866 m; 08^{0}46.912'; 037^{0}53.023'	54	MM521
90	*Rhamnus staddo* A. Rich.	Tsedo/Yedurgesho	Rhamnaceae	S	2848 m; 08^{0}46.719'; 037^{0}53.072'	52	MM550
91	*Rosa abyssinica* Lendley.	Enqoto /Kega	Rosaceae	S	2953 m; 08^{0}46.964'; 037^{0}53.813'	3	MM477
92	*Rubus apetalus* Poir	Goraa/ Enjorie	Rosaceae	S	2876 m; 08^{0}47.097'; 037^{0}54.022'	1	MM472
93	*Rubus niveus* Thunb.	Ejore	Rosaceae	S	2848 m; 08^{0}46.719'; 037^{0}53.072'	52	MM561

94	*Rumex nepalensis* Spreng.	Tulti/Tult	Polygonaceae	H	2866 m; 08^{0}46.614'; 037^{0}53.312'	57	MM560
95	*Rumex nervosus* Vahl.	Dhangaggoo/Embacho	Polygonaceae	S	2876 m; 08^{0}47.097'; 037^{0}54.022'	1	MM465
96	*Salvia nilotica* Jacq.	Sokoksa/Hulegeb	Lamiaceae	H	2896 m; 08^{0}46.942'; 037^{0}53.016'	25	MM518
97	*Sanicula elata* Buch. -Ham. ex D. Don.	Sanicle (Eng.)	Apiaceae	H	2842 m; 08^{0}46.809'; 037^{0}53.044'	53	MM569
98	*Satureja punctata* (Benth.) Briq.	Seseg Wukaria/ Yenbesa Chenger	Lamiaceae	H	2964 m; 08^{0}47.014'; 037^{0}53.240'	17	MM500
99	*Satureja punctata* (Benth.) Briq. subsp. *ovata*$^+$	Tosign Mesay	Lamiaceae	S	2973 m; 08^{0}47.104'; 037^{0}53.219'	10	MM489
100	*Scabiosa columbaria* L.	Yetja Zagol	Dipsacaceae	H	2973 m; 08^{0}46.963'; 037^{0}53.638'	5	MM481
101	*Schefflera volkensii* (Engl.) Harms	Qustya	Araliaceae	T	2860 m; 08^{0}46.656'; 037^{0}52.964'	33	MM546
102	*Senecio inornatus* DC.	Kitelesefi nechu	Asteraceae	H	2876 m; 08^{0}47.097'; 037^{0}54.022'	1	MM462
103	*Senecio ochrocarpus*$^+$ Oliv. & Hiern	Keello/Kebkebo	Asteraceae	H	3015 m; 08^{0}47.203'; 037^{0}52.919'	30	MM579
104	*Senna multiglandulosa* (Jacq.) Irwin & Barneby	Gufa/Digita mesay	Fabaceae	S	3040 m; 08^{0}46.834'; 037^{0}52.765'	24	MM516
105	*Sida schimperiana* Hochst. ex A. Rich.	Kote harree/Chifrig	Malvaceae	S	2866 m; 08^{0}46.912'; 037^{0}53.023'	54	MM523
106	*Silene burchellii* DC. form D	Wegert mesay	Caryophyllaceae	H	3044 m; 08^{0}47.132'; 037^{0}52.803'	64	MM528
107	*Solanecio gigas*$^+$ (Vatke) C. Jeffrey	Osole/Yeshikoko gomen	Asteraceae	S	2876 m; 08^{0}46.813'; 037^{0}53.244'	14	MM495
108	*Solanum marginatum* L.f.	Hidi/Geber embuay	Solanaceae	S	2856 m; 08^{0}46.704'; 037^{0}53.000'	31	MM548
109	*Solanum nigrum* L. subsp. *nigrum*	Muiulo/Tikur Awut	Solanaceae	H	2964 m; 08^{0}47.014'; 037^{0}53.240'	17	MM498
110	*Solanum villosum* Mill.	Awut	Solanaceae	H	2888 m; 08^{0}47.028'; 037^{0}52.983'	26	MM575
111	*Sporobolus angustifolius* A.Rich.	Tef aflot	Poaceae	H	2934 m; 08^{0}46.678'; 037^{0}52.962'	59	MM537
112	*Stachys aculeolata* Hook.f.	Tut Astay mesay	Lamiaceae	H	2953 m; 08^{0}46.964'; 037^{0}53.813'	3	MM567
113	*Stephania abyssinica* (Dillon & A. Rich.) Walp.	Hidda Hantuuta/ Yayit Hareg	Menispermaceae	H	2934 m; 08^{0}46.678'; 037^{0}52.962'	59	MM554

114	*Streblochaete longiarista* (A. Rich.) Pilg.	Chogogit sar	Poaceae	H	2848 m; 08^{0}46.719'; 037^{0}53.072'	52	MM549
115	*Tacazzea apiculata* Oliv.	Anano/Tefrina	Asclepiadaceae	L	2964 m; 08^{0}46.790'; 037^{0}53.065'	18	MM504
116	*Tanacetum cinarariifolium* (Trev.) Sch. Bip.	Dibilal mesay	Asteraceae	H	2896 m; 08^{0}46.942'; 037^{0}53.016'	25	MM529
117	*Thymus schimperi*$^{++}$ Ron. subsp. *schimperi*	Tosign	Lamiaceae	H	2876 m; 08^{0}47.097'; 037^{0}54.022'	1	MM459
118	*Torilis arvensis* (Hudson) Link	Spreading hedge parsley (Eng)	Apiaceae	H	2981 m; 08^{0}46.845'; 037^{0}52.801'	23	MM529
119	*Urera hypselodendron* A. Rich.	Langusto/Lanqsh	Urticaceae	L	2867 m; 08^{0}46.594'; 037^{0}52.894'	34	MM547
120	*Urtica simensis*$^{+}$ L.	Dobbi/Sama	Urticaceae	H	2866 m; 08^{0}46.614'; 037^{0}53.312'	12	MM492
121	*Verbascum sinaiticum* Benth.	Etsedebitera/ketetina	Scrophulariaceae	H	3061 m; 08^{0}46.837'; 037^{0}52.667'	60	MM553
122	*Vernonia leopoldi*$^{+}$ (Sch.Bip. ex Walp.) Vatke	Soyama/Chibo	Asteraceae	H	2980 m; 08^{0}47.002'; 037^{0}53.367'	64	MM557
123	*Vernonia myriantha* Hook. f.	Reejii	Asteraceae	S	2856 m; 08^{0}46.704'; 037^{0}53.000'	31	MM531
124	*Vernonia sp.*	Reejii/Gujo	Asteraceae	S	3015 m; 08^{0}47.203'; 037^{0}52.919'	30	MM513
125	*Vernonia urticifolia* A. Rich.	Soyama	Asteraceae	H	3044 m; 08^{0}47.132'; 037^{0}52.803'	64	MM563
