# Supplementary material for: Carbon stocks of above- and belowground tree biomass in Kibate Forest around Wonchi Crater Lake, Central Highland of Ethiopia
Source: PLoS One. 2021 Jul 9;16(7):e0254231. doi: 10.1371/journal.pone.0254231 (PMC8270183; doi:10.1371/journal.pone.0254231)
Supplement: S2 Table — AGB = Aboveground biomass, BGB = Belowground biomass, TB = Total biomass, AGC = Aboveground carbon stock, BGC = Belowground carbon stock, TC = Total carbon stock, HI = Human impact, and LG = Livestock grazing. (PDF) [file pone.0254231.s002.pdf]

S2 Table. Environmental variables, tree biomass and C stock (Mg ha^{-1}) within 66 plots in Kibate Forest. AGB = Aboveground biomass, BGB = Belowground biomass, TB = Total biomass, AGC = Aboveground carbon stock, BGC = Belowground carbon stock, TC= Total carbon stock, HI = Human impact, LG = Livestock grazing.

Plot No.	Latitude	Longitude	Altitude	Slope	Aspect	Location	AGB	BGB	TB	AGC	BGC	TC	HI	LG
1	8.781347	37.90155	2838	25	NE	Lake margin	107.02	21.40	128.42	53.51	10.7	64.21	1	1
2	8.782683	37.90018	2876	33	NE	Lake margin	119.80	23.96	143.76	59.9	11.98	71.88	0	0
3	8.784475	37.89883	2940	40	NE	Lake margin	106.64	21.32	127.96	53.32	10.66	63.98	1	1
4	8.783182	37.89709	2953	42	NE	Lake margin	138.58	27.72	166.3	69.29	13.86	83.15	0	0
5	8.782959	37.89522	2958	25	NE	Lake margin	33.38	0.84	34.24	16.69	0.42	17.12	2	4
6	8.783404	37.8934	2973	53	NE	Lake margin	101.30	20.26	121.54	50.65	10.13	60.77	1	1
7	8.784178	37.89097	2981	68	NE	Lake margin	106.50	21.30	127.8	53.25	10.65	63.9	1	1
8	8.784899	37.88958	2989	63	NE	Lake margin	142.50	28.50	171	71.25	14.25	85.5	0	0
9	8.787667	37.88643	2981	65	NE	Lake margin	68.08	13.62	81.68	34.04	6.81	40.84	1	2
10	8.78852	37.88439	2981	65	NE	Lake margin	73.28	14.66	87.94	36.64	7.33	43.97	2	1
11	8.78985	37.88288	2981	65	NE	Lake margin	3.84	0.76	4.62	1.92	0.38	2.31	3	4
12	8.779534	37.89835	2838	22	NE	Road margin	76.66	15.34	91.98	38.33	7.67	45.99	2	0
13	8.780181	37.89726	2899	35	NE	Road margin	76.28	2.28	78.54	38.14	1.14	39.27	2	2
14	8.780414	37.896	2896	36	NE	Road margin	43.24	26.18	69.42	21.62	13.09	34.71	2	2

15	8.780701	37.89467	2910	42	NE	Road margin	37.76	7.56	45.32	18.88	3.78	22.66	3	2
16	8.780987	37.89299	2924	38	NE	Road margin	73.96	14.80	88.74	36.98	7.4	44.37	2	0
17	8.781411	37.89141	2946	45	NE	Road margin	136.38	27.28	163.64	68.19	13.64	81.82	0	0
18	8.778241	37.88539	2864	24	SE	Small stream	83.32	16.66	99.98	41.66	8.33	49.99	2	0
19	8.778824	37.88441	2902	32	SE	Small stream	111.54	22.30	133.86	55.77	11.15	66.93	0	1
20	8.779428	37.88346	2910	35	SE	Small stream	33.38	6.68	40.06	16.69	3.34	20.03	3	2
21	8.780005	37.88331	2931	40	SE	Small stream	68.32	13.66	81.98	34.16	6.83	40.99	1	2
22	8.781125	37.88232	2947	43	SE	Small stream	136.68	27.34	164.02	68.34	13.67	82.01	0	0
23	8.781655	37.88143	2981	48	SE	Small stream	119.86	23.98	143.84	59.93	11.99	71.92	0	0
24	8.782196	37.88044	3016	55	SE	Small stream	76.22	15.24	91.46	38.11	7.62	45.73	2	0
25	8.782196	37.88986	2956	48	NE	Road margin	26.38	5.28	31.66	13.19	2.64	15.83	3	3
26	8.782482	37.88885	2961	48	NE	Road margin	35.24	7.04	42.28	17.62	3.52	21.14	3	2
27	8.783351	37.88769	2977	53	NE	Road margin	120.54	24.10	144.66	60.27	12.05	72.33	0	0
28	8.784889	37.88522	2973	52	NE	Road margin	78.44	15.68	94.14	39.22	7.84	47.07	0	2
29	8.786151	37.88432	2980	61	NE	Road margin	60.64	12.12	72.78	30.32	6.06	36.39	2	1
30	8.787306	37.88297	2975	58	NE	Road margin	28.18	5.64	33.82	14.09	2.82	16.91	3	2
31	8.777711	37.88208	2848	22	NW	Walga stream	105.04	21.00	126.06	52.52	10.5	63.03	1	1
32	8.777997	37.8811	2858	28	NW	Walga stream	134.20	26.84	161.04	67.1	13.42	80.52	0	0
33	8.777583	37.88006	2860	28	NW	Walga stream	77.98	15.60	93.56	38.99	7.8	46.78	1	2

34	8.777848	37.87901	2867	30	NW	Walga stream	83.21	16.64	99.86	41.61	8.32	49.93	0	2
35	8.778135	37.87797	2888	35	NW	Walga stream	139.94	27.99	167.92	69.97	13.99	83.96	0	0
36	8.779163	37.87655	2898	40	NW	Walga stream	99.95	19.99	119.94	49.98	9.99	59.97	1	1
37	8.780117	37.87635	2905	42	NW	Walga stream	123.06	24.61	147.68	61.53	12.3	73.84	0	0
38	8.780711	37.87562	2956	45	NW	Walga stream	33.90	6.78	40.68	16.95	3.39	20.34	3	2
39	8.781825	37.8749	2955	45	NW	Walga stream	100.33	20.07	120.4	50.17	10.03	60.2	1	1
40	8.782397	37.87391	2973	53	NW	Walga stream	88.25	17.65	105.9	44.13	8.83	52.95	1	2
41	8.783394	37.87289	2972	53	NW	Walga stream	11.28	2.26	13.54	5.64	1.13	6.77	3	4
42	8.783054	37.87189	2988	63	NW	Walga stream	89.57	17.91	107.48	44.78	8.96	53.74	0	2
43	8.782652	37.87948	3035	62	SE	Small stream	17.35	3.47	20.82	8.68	1.74	10.41	3	3
44	8.782333	37.87826	3040	60	SE	Small stream	24.82	4.96	29.78	12.41	2.48	14.89	3	3
45	8.783118	37.87094	2996	68	SW	Walga stream	52.74	10.55	63.28	26.37	5.27	31.64	2	2
46	8.782948	37.87006	3036	70	SW	Walga stream	80.88	16.18	97.06	40.44	8.09	48.53	2	0
47	8.783076	37.86907	3060	69	SW	Walga stream	106.14	21.23	127.38	53.07	10.61	63.69	1	1
48	8.783203	37.86813	3073	68	SW	Walga stream	24.92	4.98	29.9	12.46	2.49	14.95	3	3
49	8.7835	37.86703	3069	70	SW	Walga stream	41.53	8.31	49.84	20.76	4.15	24.92	3	1
50	8.783669	37.86556	3078	70	SW	Walga stream	39.54	7.91	47.46	19.77	3.95	23.73	3	1
51	8.776661	37.89428	2811	20	NE	Middle forest	77.80	15.56	93.36	38.90	7.78	46.68	1	2
52	8.777477	37.89277	2848	25	NE	Middle forest	87.78	17.56	105.34	43.89	8.78	52.67	0	2

53	8.777795	37.89183	2842	22	NE	Middle forest	107.99	21.60	129.58	53.99	10.80	64.79	1	0
54	8.778188	37.89084	2866	30	NE	Middle forest	105.05	21.01	126.06	52.52	10.50	63.03	1	1
55	8.778654	37.88983	2860	28	NE	Middle forest	47.15	9.43	56.58	23.57	4.71	28.29	2	2
56	8.779195	37.88876	2915	38	NE	Middle forest	88.67	17.73	106.4	44.33	8.87	53.2	1	2
57	8.779577	37.88772	2921	42	NE	Middle forest	39.32	7.86	47.18	19.66	3.93	23.59	2	3
58	8.780022	37.88667	2932	43	NE	Middle forest	58.00	11.60	69.6	29.00	5.80	34.8	2	2
59	8.78033	37.88575	2934	43	NE	Middle forest	67.44	13.49	80.92	33.72	6.74	40.46	1	2
60	8.780648	37.88467	2959	48	NE	Middle forest	81.19	16.24	97.44	40.60	8.12	48.72	0	2
61	8.781634	37.88408	2964	52	NE	Middle forest	78.39	15.68	94.06	39.19	7.84	47.03	0	2
62	8.782143	37.88321	2979	55	NE	Middle forest	83.10	16.62	99.72	41.55	8.31	49.86	1	2
63	8.782768	37.88237	3021	66	NE	Middle forest	52.74	10.55	63.28	26.37	5.27	31.64	2	2
64	8.783903	37.88164	3044	68	NE	Middle forest	93.17	18.63	111.8	46.58	9.32	55.9	1	1
65	8.784507	37.88099	3056	69	NE	Middle forest	39.54	7.91	47.46	19.77	3.95	23.73	3	1
66	8.785832	37.88078	3074	70	NE	Middle forest	47.01	9.40	56.42	23.51	4.70	28.21	2	2
